# Single molecule sequencing of the M13 virus genome without amplification

**DOI:** 10.1371/journal.pone.0188181

**Published:** 2017-12-18

**Authors:** Luyang Zhao, Liwei Deng, Gailing Li, Huan Jin, Jinsen Cai, Huan Shang, Yan Li, Haomin Wu, Weibin Xu, Lidong Zeng, Renli Zhang, Huan Zhao, Ping Wu, Zhiliang Zhou, Jiao Zheng, Pierre Ezanno, Andrew X. Yang, Qin Yan, Michael W. Deem, Jiankui He

**Affiliations:** 1 Direct Genomics Co., Ltd., Shenzhen, Guangdong, China; 2 Reproductive Medical Center of Guangdong General Hospital & Guangdong Academy of Medical Sciences, Guangzhou, China; 3 Shenzhen Armed Police Hospital Reproductive Center, Luohu District, Shenzhen, China; 4 Department of Biology, South University of Science and Technology of China, Shenzhen, Guangdong, China; 5 Departments of Bioengineering and Physics & Astronomy, Rice University, Houston, TX, United States of America; Harbin Institute of Technology Shenzhen Graduate School, CHINA

## Abstract

Next generation sequencing (NGS) has revolutionized life sciences research. However, GC bias and costly, time-intensive library preparation make NGS an ill fit for increasing sequencing demands in the clinic. A new class of third-generation sequencing platforms has arrived to meet this need, capable of directly measuring DNA and RNA sequences at the single-molecule level without amplification. Here, we use the new GenoCare single-molecule sequencing platform from Direct Genomics to sequence the genome of the M13 virus. Our platform detects single-molecule fluorescence by total internal reflection microscopy, with sequencing-by-synthesis chemistry. We sequenced the genome of M13 to a depth of 316x, with 100% coverage. We determined a consensus sequence accuracy of 100%. In contrast to GC bias inherent to NGS results, we demonstrated that our single-molecule sequencing method yields minimal GC bias.

## Introduction

The sequencing of the human genome [1, 2] and the ensuing development of next-generation sequencing technologies (NGS) has revolutionized the life sciences and brought new approaches to applications as diverse as pathogen detection [3, 4], forensics [5, 6], and clinical diagnosis [7,8]. The advent of precision medicine [9] promises profound advances in the clinic, leveraging sequencing results for diagnosis of cancer [10, 11] and inherited disease [12, 13]. Despite the advantages of NGS platforms, the costly, time-intensive process of NGS sample library preparation and the use of polymerase chain reaction (PCR) amplification limit the efficiency and practicality of NGS in the clinic.

The preparation of DNA libraries in NGS generally requires a preliminary step based on PCR amplification. This process introduces bias and can result in incorrect interpretation of raw data [14, 15]. The popular Illumina sequencing platform produces data sets with uneven coverage and serious defects in GC-poor or GC-rich regions. Low coverage regions could be interpreted as sequencing errors by most current assemblers [16], and high coverage regions could be interpreted as repetitive sequences [17, 18], introducing hard-to-correct errors in NGS results. Much effort has gone into improving protocols for NGS library preparation to reduce or fully suppress GC bias [19, 20]. Single-molecule (SM) sequencing circumvents these library preparation issues by avoiding PCR amplification altogether.

First proposed in 1989 [21], SM sequencing is now seen as the successor to NGS [22] in the progression of sequencing technology development. Different SM sequencing technologies have rapidly developed over the past decade, with progress on read length, sequencing time, and data throughput. Three technologies are now well known, each with their unique characteristics: (i) the first true single molecule sequencing (tSMS) combined with sequencing-by-synthesis (SBS) [23] technology from Helicos Biosciences [24, 25]; (ii) single molecule real time (SMRT) sequencing technology from Pacific Biosciences producing super long read length (longer than 10k bases [26, 27]), but relatively low throughput; and (iii) Oxford Nanopore Technologies, producing long read length (6k bases [26]) but limited accuracy and low throughput. The GenoCare platform improves on principles from the Helicos Biosciences platform.

A combination of minimal, amplication-free sample preparation and efficient massively parallel short reads processing are ideal for the demands of sequencing-based clinical diagnosis. Advantages of GenoCare SM sequencing include (i) a simple and time-saving sample preparation consisting of DNA shearing followed by poly-A tailing and 3' end blocking steps, (ii) absence of PCR amplification and its associated base substitution errors, and (iii) potential for RNA SM sequencing for investigation of transcriptomic aspects of gene expression.

Our approach is devised to provide simple operation and high-throughput, unbiased data. Recently, we demonstrated a direct targeted sequencing of cancer related gene mutations at the SM level [28]. In this study, we describe the performance of our new GenoCare platform for SM sequencing without preliminary PCR amplification.

## Results

### Sequencing process

**Fig 1:**
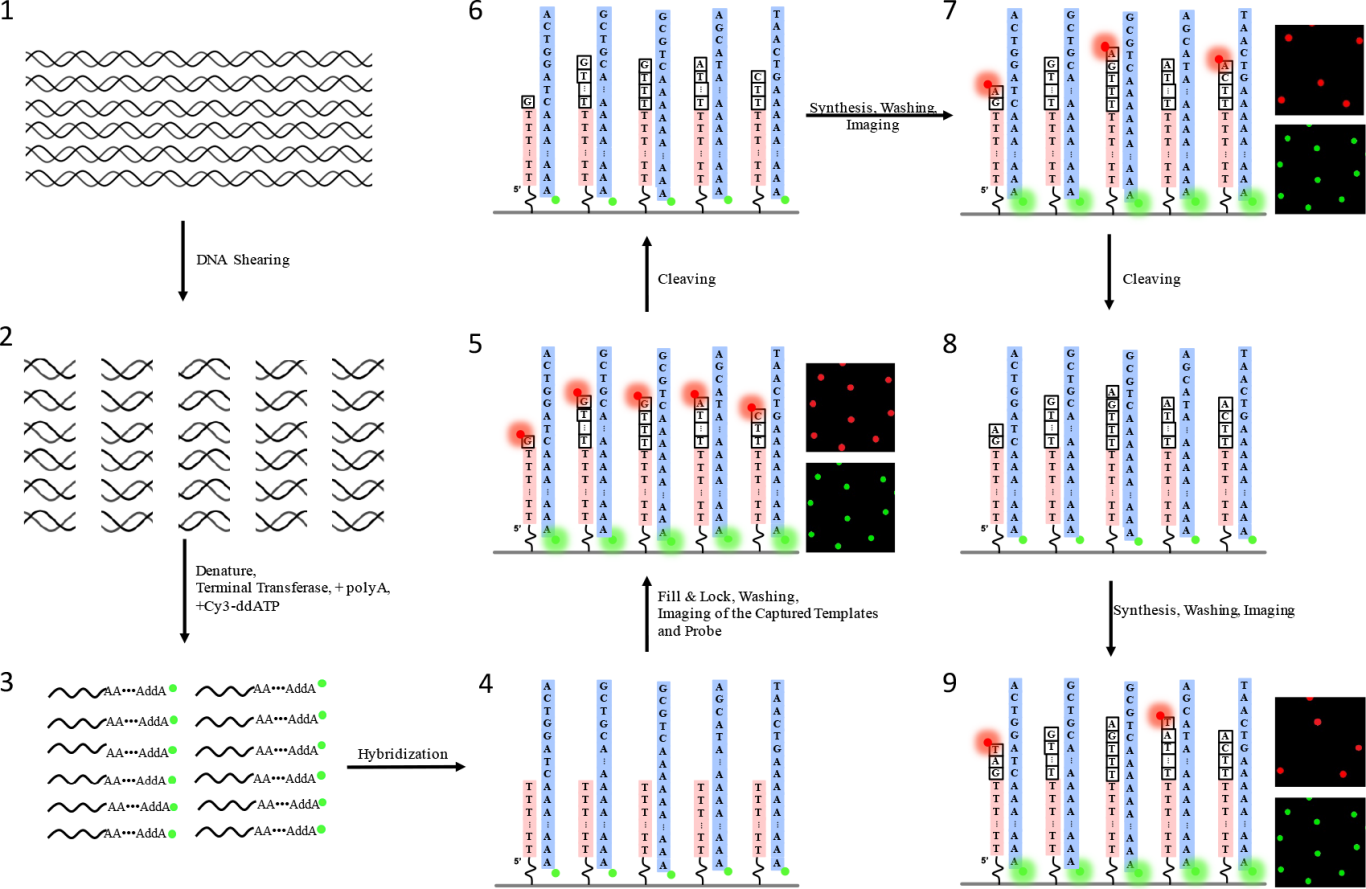
Sample preparation and sequencing process for single molecule sequencing of biological samples.

Our SBS scheme is shown in **Fig 1**. Sample preparation is simple, fast, and amplification-free. M13 genomic DNA was sheared into fragments of ~200bp, poly-A tailed with tail length of 50-100nt, and blocked by ddATP-Cy3. Sequencing surfaces were chemically modified and covalently bound with poly(T) oligonucleotides, allowing for hybridization with target DNA. Once annealed, residual dATP were filled with natural nucleotides, and locked with one reversible terminator.

The single molecule SBS process has been described previously [28]. Each cycle includes terminator incorporation, imaging, fluorophore cleavage, and residual bond capping. The GenoCare platform adopts total internal fluorescence microscopy (TIRF) to observe single molecules. Integration time of 200 ms guaranteed a good signal-to-noise ratio and reduced the photobleaching of dyes. Just 0.5% of one flow-cell channel was needed to resequence the M13 virus genome. We sequenced 80 cycles (20 quads of CTAG), and analyzed the images to perform base-calling (**S1 File and S1 Scheme**). Sequence data was uploaded to NCBI Sequence Read Archive (SRA) with accession number SRR6168186. Sample preparation took 3 hours and instrument run time was 9 hours.

### Genome coverage

104,802 reads were uniquely aligned to the reference genome, accounting for 25.4% of the total reads. Reads matching the following criteria were discarded: 1) reads shorter than 13 bases after alignment, 2) reads including a sequence exactly matching the terminator addition order, indicating non-specific adsorption, and 3) reads mapped to multiple locations on the reference genome. Among mapped reads, the dominant error was deletion (1.65%), followed by insertion (0.78%) and substitution (0.69%) (**Table 1**). We calculated the error rates in homopolymer regions and non-homopolymer regions. Homopolymer was defined as 3 or more identical bases in a row. The results show that, in homopolymer regions, substitution error rate is 1.23%, followed by insertion 1.04% and deletion 0.86%. In non-homopolymer regions, error rates are like substitution 0.60%, insertion 0.71% and deletion 1.84%. Relatively low deletion rate in homopolymer regions indicates satisfactory blocking efficiency. Considering that a deletion followed by an insertion can also be called as a substitution, we looked at the total error rate (3.13% vs 3.15%), which demonstrates the lack of homopolymer issue using our method [29].

**Table 1:** M13 genome sequencing statistics.

Cycles Sequenced	Average Read length	Coverage			Total Reads	Mapped Reads	Unique Mapped Reads	Unique Mapped Ratio	Sub Rate	Del Rate	Ins Rate
		Average	Max.	Min.							
80	22 base	316x	717x	18x	409491	104802	103990	25.4%	0.69%	1.65%	0.78%

**Fig 2:**
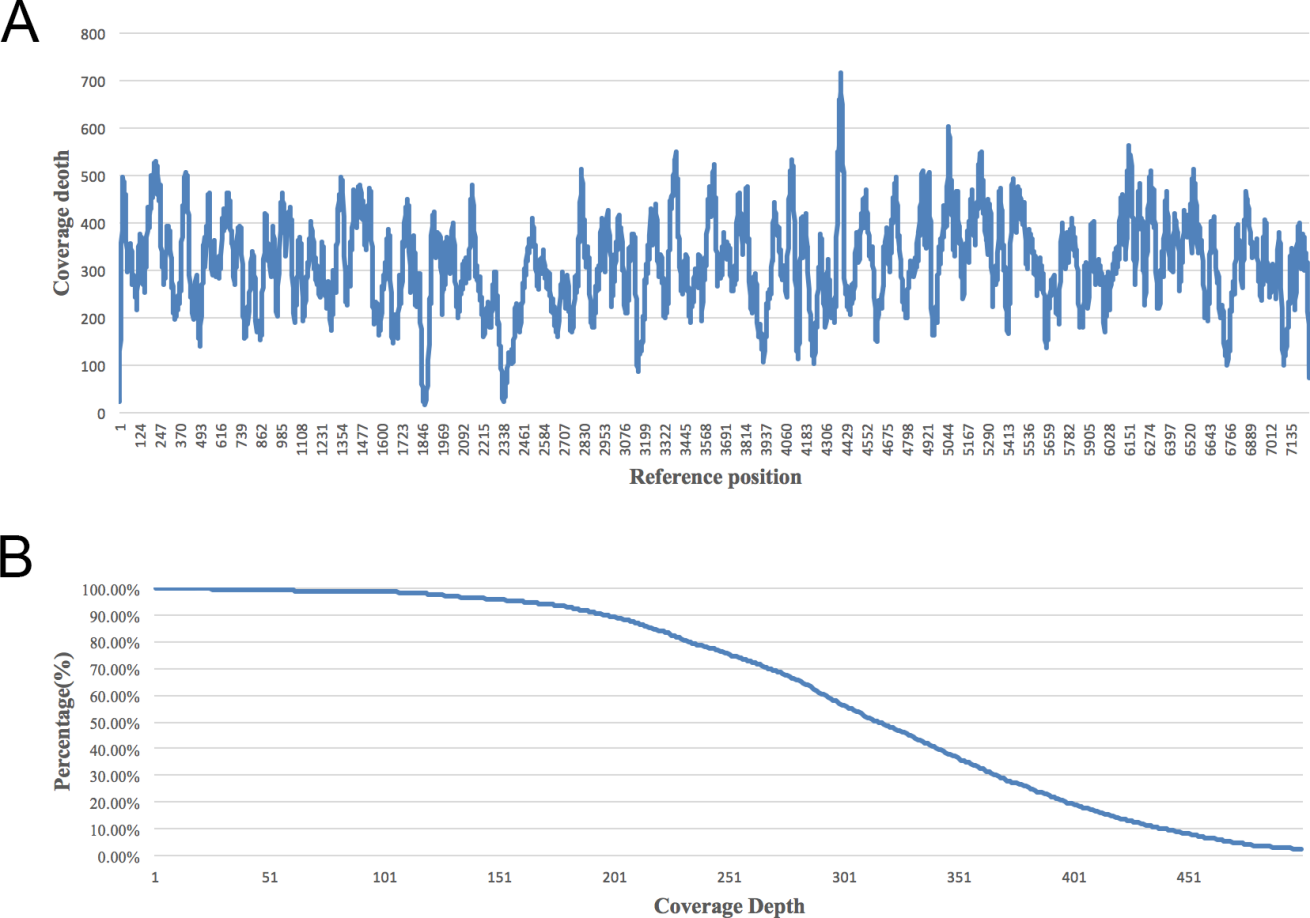
Coverage depth distribution. (A) Coverage depth for each base on M13 reference. The average coverage depth is 316x±96x. (B) Coverage rate as a function of coverage depth.

Most reads (53,100) aligned perfectly to the reference with no errors, and aligned reads had at most 3 errors, as specified by our alignment algorithm (**S1 Fig**). The average coverage depth for each base was 316x, and the minimum coverage was 18x (**Fig 2A**). The variation in coverage depth is due to several reasons: 1) Non-random fragmentation by DNase. 2) Non-unique mapped reads were filtered which may cause lower coverage depth. Abnormal GC content also contributes to low coverage. 3) In M13 genome, there are some areas that contain sequences similar to the base addition order, which may artificially increase the coverage because of non-specific adsorption. The coverage depth profile can be seen in **Fig 2B**. The Integrative Genomics Viewer (IGV) gives a clear picture of mapping against the known M13 genome reference (**S2 Fig**).

### Read length

**Fig 3:**
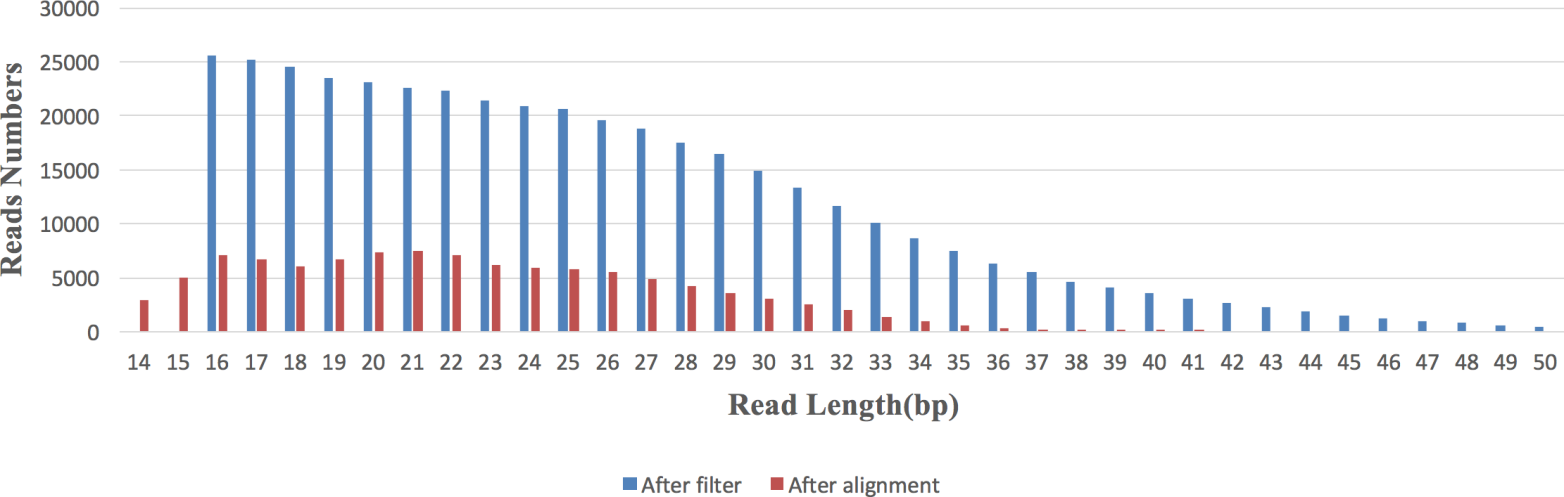
Read length distribution after length and repeat filters (blue bars) and after alignment (red bars).

Read length for this M13 sequencing run is shown in **Fig 3**. After conducting 80 base incorporation cycles and filtering, the average read length was 22 bases (**Table 1**). Before filtering, a peak was observed in the read length distribution at 25 bases.

### GC bias

**Fig 4:**
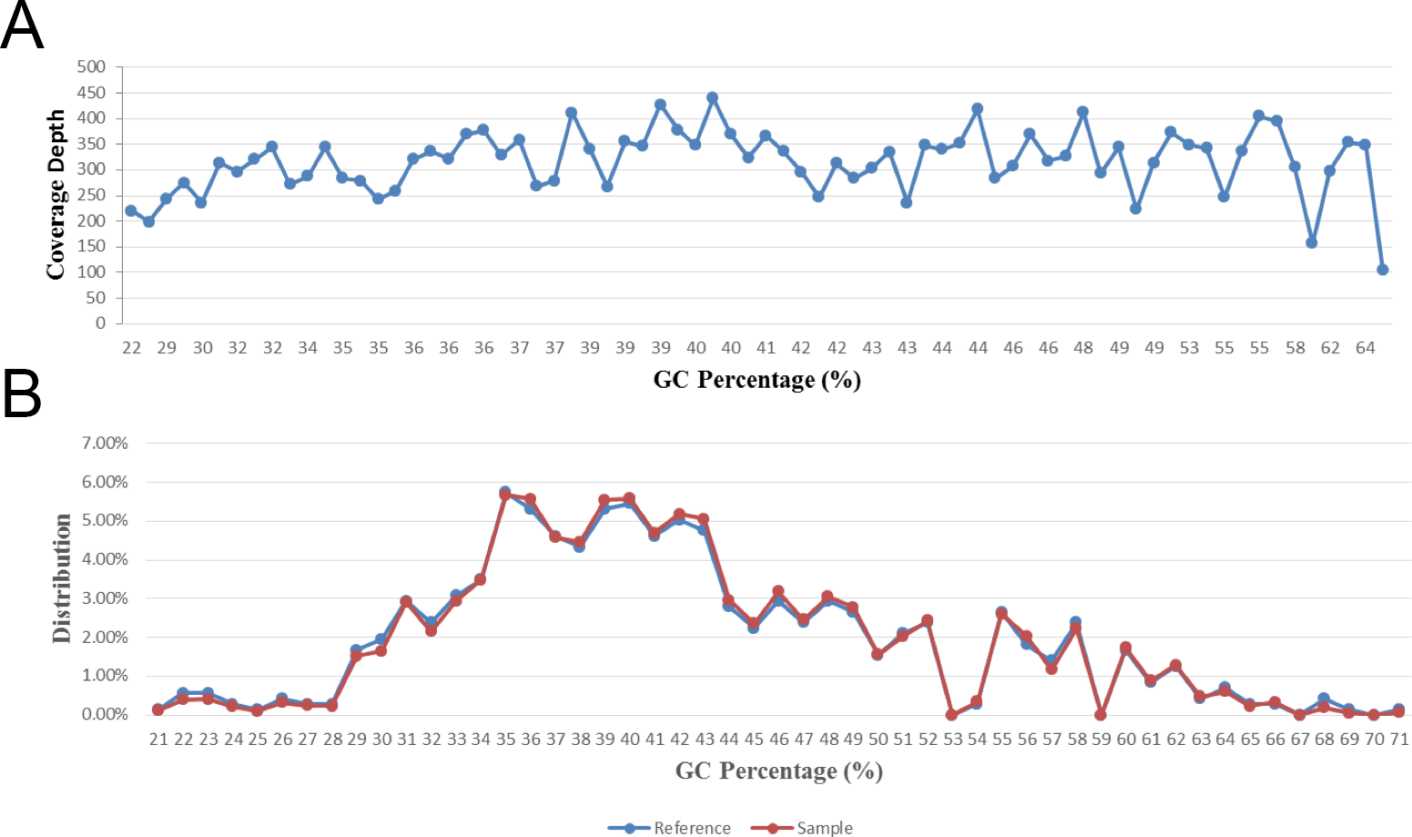
GC content. (A) Average depth distribution of all 100-base windows as a function of GC content. From GC content 22% to 69%, the average depth of each window in the genome fluctuates in a small range. (B) GC patterns of the reference genome and aligned reads.

No obvious GC bias was observed in the coverage depth of 100 base windows over a GC content range of 22–69% (**Fig 4A**). The distribution of base frequency in the reference as function of the GC content shows an almost identical shape to the depth distribution calculated from the sequencing result (**Fig 4B**). The R^2^ (goodness of fit) of those two curves is 0.9946, indicating minimal coverage bias observed in this experiment.

## Discussion

### Alignment profile

The cloning vector M13mp18 was sequenced on this new GenoCare platform. Similar deletion rates in homopolymer (1.87%) and non-homopolymer (1.46%) regions demonstrate reasonable blocking efficiency by the terminators. For the 7.2 kb M13 genome, the average read length of 22 bases was adequate for alignment. Before alignment, the length distribution showed a peak at 25 bases. Filtering high-error and non-uniquely mapped reads lowered throughput and average read length. The reported average read length can also be attributed to the relatively small number of cycles run (80); thus there is potential for longer read length on the GenoCare platform as cycle number is increased in future experiments. As predicted from the absence of PCR amplification in our platform, we observed minimal GC bias in this experiment, demonstrating a key advantage of SM sequencing over NGS.

### Clinical applications

In this study, we demonstrated the new GenoCare platform’s SM sequencing capabilities. Overall sequencing took 12 hours including sample preparation, instrument run time and data analysis—a major improvement over NGS standards. This reduction in sequencing time is of great importance in the clinic, where timely results and diagnosis are critical. In sequencing the M13 genome, GenoCare used only 0.5% of one flow cell channel. Thus, GenoCare is capable of vastly increased throughput and has potential for whole human genome sequencing. Because our platform uses poly-T oligonucleotides to hybridize with poly-A tailed DNA, there is potential for GenoCare to handle naturally poly-A tailed RNA, and address needs for new technologies in transcriptomics. GenoCare is an automated desktop sequencer for dedicated use in the clinic, eclipsing NGS technologies with the potential to deliver faster and cheaper sequencing results with limited GC bias.

## Materials and methods

### Sample preparation

M13 genomic DNA preparation process was illustrated in **Fig 1**.

#### M13

M13mp18 cloning vector was purchased from NEB, Beijing, China, and used as received. The sequence of the M13mp18 cloning vector is derived from the M13 phage [30] and contains 7249 bp. In this study, we used this cloning vector as DNA raw material to re-sequence, analyze, and compare with the reference sequence.

#### Oligonucleotide primers

5’ amine functionalized Poly-T oligonucleotides were purchased from Sangon and used as received.

#### DNA fragmentation

The M13mp18 cloning vector (from NEB, ref. N4018S) was used as raw DNA material to be sequenced by our platform. This cloning vector was first randomly fragmented into dsDNA fragments of about 200 bp using NEBNext® dsDNA Fragmentase® (from NEB, ref M0348S). Then, DNA fragments were purified using Agencourt AMPure XP beads (from Beckman, ref. A63881). The concentration of DNA was assessed by UV absorption using a Nanodrop 2000 device.

#### Poly-A tailing and blocking

Multiple incorporations of 50–100 dATP at the 3' end of ssDNA fragments from the cloning vector resulted in a poly-A tail. This reaction completed within 20 minutes. In a second step, poly-A tailed 3' ends were blocked by incorporating the Cyanine 3 dideoxy ATP (Cy3-ddATP from PERKINELMER, ref. NEL586001EA). The blocking reaction completed within 30 minutes using the enzyme Terminal Transferase (from NEB, ref. M0315) such that the incorporation of reversible terminators at the 3' end of the template strands was prevented.

### Surfaces and template capture

#### Surface chemistry

Sequencing surfaces were prepared on 110×74 mm epoxy-coated glass coverslips (SCHOTT, Jena, Germany). Poly-T oligonucleotides were covalently bond to surface.

#### Flow cells

The above functionalized glass coverslip was assembled with a 1.0 mm thick glass slide by a pressure sensitive adhesive to form a flow cell. The flow cell has 16 channels, determined by the adhesive shape. For the M13 sequencing in this experiment, ~0.5% of one channel was imaged.

#### Template capture (hybridization)

The surface of the flow-cell was chemically modified by anchoring poly-T ssDNA strands at their 5' end, in order to capture poly-A tailed strands from the library once they were injected inside the flow-cell at 55°C. Then non-hybridized templates were washed away by 150 mM HEPES, 1X SSC and 0.1% SDS, followed by 150 mM HEPES and 150 mM NaCl.

### Sequencing reactions

#### The GenoCare platform

All the sequencing reactions were implemented on the GenoCare platform The GenoCare is an automated single molecule sequencer with three major components: fluorescence imaging system, microfluidic system, and the stage to control the movement of sample. The imaging system is based on total internal reflection fluorescence (TIRF) microscopy [28]. GenoCare is designed for clinical applications and it outperforms the previous platform developed by Harris et al [29] in terms of read lengths, coverage depth, error rate and sequencing time.

#### Fill and lock

Because the hybridization of poly-T primer with poly-A tailed template may not be perfect, a step to fill the remaining dATP on the template with dTTP before the real sequencing process starts is necessary. After hybridization, the temperature of the flow-cell was lowered to 37°C. The unpaired adenine nucleotides of poly-A tailed template strand were paired by multiple incorporations of natural thymine nucleotides at the 3' end of primer strands. A mixture of dATP, dCTP, and dGTP reversible terminators were added to block further incorporation so that the template was locked in place and ready for sequencing.

#### Nucleotide addition

Reversible terminators were adopted in the sequencing-by-synthesis approach. They are modified nucleotides, which are composed of nucleotide triphosphates, a fluorophore (Atto647N), disulfide linker, and an inhibitor group. The design of the inhibitor effectively blocks the incorporation of next nucleotide before cleavage of previous reversible terminator’s disulfide bond.

The DNA extension was carried out at 37°C in Tris buffer containing polymerase, one of the four nucleotides and other salts. The components of this system are available with the use instructions from Direct Genomics.

## Supporting information

S1 File Description of imaging analysis process. (DOCX)

S1 Scheme Imaging processing flow chart. (TIF)

S1 Fig Error distribution for all unique mapped reads. Most of those reads have zero or one error.
(TIF)

S2 Fig Part of an IGV view of mapping. The sequence at the bottom is the reference sequence. Capital letters show the mismatch sites, black horizontal lines indicate deletion errors, and purple vertical lines denote insertions.
(TIF)
