# Supplementary material for: Single molecule sequencing of the M13 virus genome without amplification
Source: PLoS One. 2017 Dec 18;12(12):e0188181. doi: 10.1371/journal.pone.0188181 (PMC5734777; doi:10.1371/journal.pone.0188181)
Supplement: S1 File — (DOCX) [file pone.0188181.s001.docx]

**IMAGING ANALYSIS**

The image processing algorithm shown in **Scheme S1** is designed specifically for single molecule sequencing technique and can be divided into 9 steps [1, 2].

**Step 1: Image Denoising**. Use morphological white top-hat method to subtract image background.

**Step 2: Binary Image**. Use a threshold to realize the binaryzation of image $BI(x,y)=\left\{ \begin{matrix} 0 if f(x,y)/g\left( x,y \right)<threshold \\ 1 if f(x,y)/g\left( x,y \right)\geq threshold \end{matrix} \right.$

$f(x,y)$ denotes the signal of the image, and $g\left( x,y \right)$ is the background of the image.

**Step 3: Mexican Hat Filter**. Apply Mexican Hat kernel on denoised image, and use OSTU method to get threshold *T*;

**Step 4: Spots Detection**. A two-steps strategy was adopted. First, pixels whose intensity was higher than their 8 adjacent points were selected as candidate spots. Second, candidate spots which

$$I_{max}\left( x,y \right)*A_{BI}\left( x,y \right)*{ceof}_{guass}\left( x,y \right)>T | \left( x,y \right)\in D$$

Were regarded as real signal spot, where $I_{max}\left( x,y \right)$ is the intensity of center of $m*m$ window, $A_{BI}\left( x,y \right)$ is the ratio of pixels with value 1 in the $m*m$ window for $BI(x,y)$ image, and ${ceof}_{guass}\left( x,y \right)$ is the correlation coefficient between $m*m$ window and the 2-D Gaussian template function;

**Step 5: Image Registration**. Phase-only Correlation (POC) function was applied to register images on frequency domain;

**Step 6: Templates Statistics**. First 8 cycles were used to count all templates positions. If one signal was detected in cycles 1-4 and also detected in cycles 5-8 at the same position, the signal would be regarded as a real template;

**Step 7: Base Call**. A signal that was detected within a threshold distance near one template was called a base;

**Step 8: Reads Filter**. Three filters were applied to filter bad quality reads. First, ***length filter***: filter out reads shorter than a threshold. Second, ***repeat filter***: if one template position continuously detects a signal for $n$ cycles, the template will be regarded as an adsorbed noise. Third, ***no reaction filter***: if one template position does not detect signal for $n$ cycles, the template will be regarded as an ended template and ignored for the following cycles.

**Step 9: Local Alignment**. For alignment, bowtie2 was applied for mapping fasta file to the reference. The parameters used in this paper were "—local –D 20 –R 3 –N 0 –L 10 –i S,1,0.50".

**GC Bias Patterns.** GC-content bias describes the dependence between fragment count (read coverage or depth) and GC content found in high-throughput sequencing. In **Figure 4b** we analyze regularities in the GC-bias patterns by adopting a window-based approach [3]. This approach allows the detection of aberrations down to every GC-count of 100-base windows. For reference DNA, we denote the length of genome by L,

$$Count(GC_{i})=\sum_{j=1..L} base_{(GC=i)_{window}}\text{ i=0,1...window }$$

For experimental data,

$$Count(GC_{i})=\sum_{j=1..L} depth_{(GC=i)_{window}}\text{ i=0,1...window}$$

We provide a single position model that estimates the depth count for individual positions rather than reads. We can detect small depth aberrations in low coverage data by turning it into a GC base frequency and GC depth frequency. For each GC-count bin, we compare the normalized base frequencies to the normalized depth frequencies. Both GC rich fragments and AT rich fragments are represented. The GC effect for genomes is unimodal. In AT rich regions, coverage increases with increasing GC. In GC rich regions, coverage decreases with increasing GC. The shape of the distribution varies for different genomes. In this experiment, the experimental distribution curve overlaps perfectly with the theoretical one, indicating that there is no apparent GC bias.

**REFERENCES**

[1] Reddy BS, Chatterji BN, “An FFT-based technique for translation, rotation, and scale-invariant image registration” IEEE Trans Image Process. 1996(Aug);5(8):1266-1271

[2] Whiteford N, Skelly T, Curtis C, Ritchie ME, Löhr A, Zaranek AW et al., “Swift: primary data analysis for the Illumina Solexa sequencing platform” Bioinformatics. 2009(Sep);25(17):2194-2199

[3] Benjamini Y, Speed TP, “Summarizing and correcting the GC content bias in high-throughput sequencing” Nucleic Acids Res. 2012(May);40(10):e72-85
