# Supplementary figures and images for: Single molecule sequencing of the M13 virus genome without amplification
Source: PLoS One. 2017 Dec 18;12(12):e0188181. doi: 10.1371/journal.pone.0188181 (PMC5734777; doi:10.1371/journal.pone.0188181)

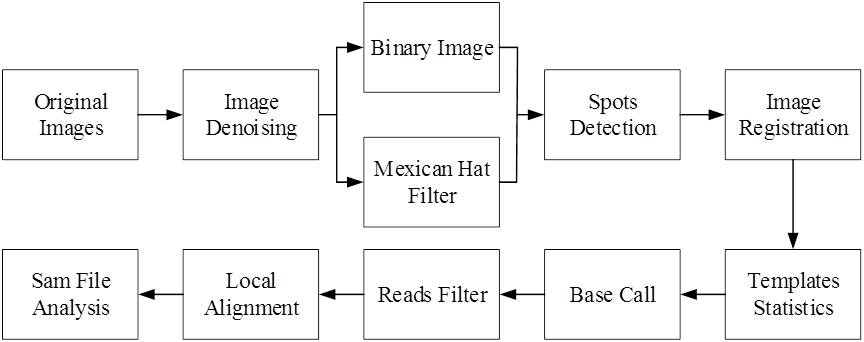

Supplement: S1 Scheme — (TIF) [file pone.0188181.s002.tif]

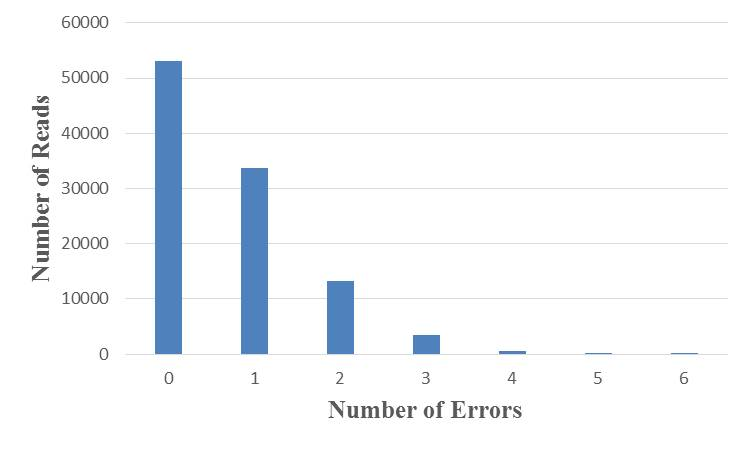

Supplement: S1 Fig — Most of those reads have zero or one error. (TIF) [file pone.0188181.s003.tif]
